# The Critical Role of Consumers in the Prevention of Foodborne Diseases: An Ethnographic Study of Italian Families

**DOI:** 10.3390/foods11071006

**Published:** 2022-03-29

**Authors:** Alessio Menini, Giulia Mascarello, Mosè Giaretta, Alice Brombin, Silvia Marcolin, Fabrizio Personeni, Anna Pinto, Stefania Crovato

**Affiliations:** Istituto Zooprofilattico Sperimentale delle Venezie, Viale dell’Università 10, 35020 Legnaro, Italy; amenini@izsvenezie.it (A.M.); mgiaretta@izsvenezie.it (M.G.); alice.brombin@unitn.it (A.B.); smarcolin@izsvenezie.it (S.M.); fpersoneni@izsvenezie.it (F.P.); apinto@izsvenezie.it (A.P.); scrovato@izsvenezie.it (S.C.)

**Keywords:** ethnography, microbiological risks, chemical risks, foodborne diseases, food handling, food safety, social research, qualitative methods, participant observation

## Abstract

A high incidence of foodborne diseases occurs in the home setting because consumers adopt inappropriate preparation, consumption, and storage procedures. The present study applies an ethnographic approach to identify inadequate practices that could increase the incidence of foodborne diseases. Techniques related to the ethnographic approach were used: participant observation, kitchens mapping, collection of photographic material, and informal interviews in natural settings. A sample of 14 families was involved through the snowball sampling technique. This study identifies habitual practices and routine behaviour as the main risk factors. The inadequacies most frequently encountered related to the microbiological risks are incorrect handwashing, the presence in the kitchen spaces of objects unrelated to food preparation, the improper use of dishcloths and sponges, the inappropriate washing of utensils and food, the incorrect storage of food in the fridge, and the presence of children and pets without an adequate administration of the spaces. The practices that can expose consumers to chemical risk include food preservation through unsuitable containers/materials, food overcooking, and detergents contamination. The data underline the need to implement communicative and training interventions that give precise and targeted indications about correct safety practices in the home setting.

## 1. Introduction

Foodborne diseases continue to be a burden on national economies and thus become a significant challenge for public health systems globally [1,2,3,4]. Despite countries’ increasing attention in managing food safety in the industrial chain, foodborne diseases continue to pose a health risk to consumers worldwide [2,4].

The World Health Organization recently estimated that 600 million—almost 1 in 10 people—fall ill after eating contaminated food, and 420,000 die every year [5]. The subjects most vulnerable to contracting foodborne diseases in our communities are individuals over 60 years old, children under the age of 5, pregnant women, and those with compromised immune systems.

Epidemiological data indicate that the risk of contracting foodborne diseases is more widespread in the home setting [1,2,6,7,8,9] and is attributable to consumers’ improper food-handling practices.

The attention to these risks has become necessary due to changing lifestyles [10,11,12,13] and increasing public awareness of health and product safety [14]. Furthermore, the social changes in work and family organisation that have taken place in the last 50 years have contributed to reducing the time available for the preparation and consumption of homemade foods, with a consequent increase in the consumption of pre-cooked and ready-to-eat foods, which in any case require extraordinary attention to handling and conservation [12,15,16,17].

Consumers generally underestimate the risks associated with foodborne diseases; first, because these risks are determined by hazards acting at levels investigated by microbiology, chemistry, and physics, which are not immediately accessible to human observers without appropriate techno-scientific instrumentation [18]. Epidemiological reports underestimate foodborne illnesses because they often generate mild symptoms that are neither reported nor detected by competent authorities [19]. Furthermore, food products pass through a very complex production network, and it is thus difficult to attribute in which node of production the contamination (microbiological or chemical) took place [17,20,21]. Generally, people tend to underestimate their chances of experiencing adverse events and fall into ‘optimistic prejudice’ or believe that they can control them [22,23,24]. However, inappropriate transport storage and handling practices at home could expose consumers to food risks. Therefore, the study of private places where food is processed and consumed, in solitude or collectively, becomes central to exploring the phenomenon of food and epidemiological risks and the conditions that favour their occurrence.

### 1.1. Subject and Aims of the Study

The ethnographic study presented here aims to detect the practices adopted by consumers that may predispose or contribute to the occurrence of a foodborne disease resulting from biological (pathogenic microorganisms) or chemical (chemical substances) risks. The main research question inspiring the study is the following: ‘To what extent are consumers’ food behaviours in the domestic environment related to risk and safety assessments?’

As part of biological risk, foodborne diseases can be infections or intoxications. Disorders are derived from viable parasites, viruses, or pathogenic bacteria in the ingested food. Intoxications occur when foods are eaten which contain toxins already formed by bacteria or by the ingestion of food with live bacteria that multiply inside the host producing toxins. Microorganisms can survive improper inactivation attempts, which usually occur with cooking, or they can be transferred indirectly by cross-contamination [25,26,27]. Microbial contamination tends to be underestimated as it falls into Beck’s model [28] as one of the ‘traditional risks’ always present in nature [29].

Conversely, chemical risk falls into the ‘modern risks’ produced by industrialisation, technologies, and human intervention [17,29]. Unlike ‘traditional’ risks, these appear to have a more significant impact, are more challenging to manage and contain, and generate most public concerns [28,29]. Chemical substances that can cause foodborne diseases can be of various natural or anthropogenic origin, unwanted cooking byproducts, and environmental pollutants due to human activity or improperly added to food [30]. Although chemical hazards depend primarily on the responsibility of producers and institutions in charge of health surveillance, they can also occur due to the problematic food-handling methods adopted by consumers.

However, both types of risk identified (microbiological and chemical) can be related to consumers’ behaviour in the domestic environment. This study identifies the factors that expose consumers to foodborne diseases by observing the behaviours and habits of a selected sample of households within their home environment during a routine time such as lunch or dinner. Watching their performance allowed to represent the contexts and situations potentially facilitating the occurrence of microbiological and chemical risks.

The present study aims to enrich the scientific and institutional debate on food safety practices in the domestic environment with social and behavioural data since this debate is often focused only on the clinical and epidemiological aspects of foodborne diseases. In this way, it is possible to plan adequate interventions focused not only on the care of individuals and groups but also through preventive and educational interventions informed by the social factors involved.

This study supports risk communication from a preventive and training perspective, trying to reduce discrepancies or inconsistencies between consumers’ knowledge and behaviours and experts’ advice [31]. This allows the refinement of the models provided by the experts, enhancing them with the multiple facets of consumers’ life contexts.

### 1.2. State of the Art

In recent decades, social studies on consumers’ risk and their eating habits have increased as a result of some food scandals [17,32]. Most studies on behaviour in the kitchen make use of quantitative social research methods to measure attitudes and perceptions regarding food risk issues (including foodborne illness), while qualitative approaches and, in particular, ethnographic ones are less used in this context [29,33,34,35,36]. However, some of these studies [21,37,38,39,40,41,42,43,44] have shown the usefulness of observational methods, both in combination with other research methods and not. Adopting observational and participatory methods applied to natural contexts entails several advantages. In the first place, they are helpful to integrate, challenge, and reinforce data that have already been obtained in other ways [45], and from a practical point of view, they allow us to go beyond the predominance of attitudinal and perceptive studies (psychometric approach). Therefore, they are valuable methods for verifying self-reported behaviours in surveys and interviews to reduce the ‘effects of the discrepancy’ and the bias of ‘social desirability’ [46,47].

The ethnographic approach is particularly suitable for studying everyday social practices. Direct observation is helpful to identify habits, attitudes, and the so-called ‘taken for granted’—the epistemological hinterland on which many routine actions are modulated unconsciously and according to mechanisms of limited rationality [48]. This also allows to grasp the behaviours that are often not recorded and that other investigation tools cannot register. Finally, this method allows the theoretical models to dialogue inductively with the empirical world, thus constructing refined representations of the investigated contexts, which pay greater attention to the complexity of reality and the contingency and contextuality of phenomena.

## 2. Methods

### 2.1. Sampling

The present study involved a selected sample of 14 households living in the Italian provinces of Padua, Bologna, and Venice and recruited through the snowball sampling technique [49]. Families with vulnerable individuals such as pregnant women, children, and people over 60 were also involved. In addition, attention was paid to the presence of pets (Table 1).

### 2.2. Method and Data Collection

Techniques related to the ethnographic approach were applied: kitchen mapping, participant observation, photographic documentation, and informal interviews conducted in the participants’ natural setting.

The ethnographic observations were conducted in October and November 2019, mainly at dinner or lunchtime depending on the availability of the participants, with an average duration of about two hours. Participants prepared a typical lunch or dinner, which generally involves all family members or people who usually frequent the house (e.g., grandchildren, grandparents, and friends). The selected families were asked to fill in a form relating to the personal data of the family members and to sign a specific, informed consent form relating to the use of the data and the methods of participation. Finally, at the end of the observation, each family received a 50 euros voucher to purchase books and music as an incentive.

The collection and analysis of ethnographic data were designed based on a socio-anthropological methodology, with the adoption of a ‘grounded approach’ [50]. Proceeding inductively, data collection started from the practices implemented by the participants and reached more general behavioural models: their culture [51] and epistemologies; in other words, how single individuals, or the groups to which they belong, know, think, and decide [52].

Each family was visited by two observers who collected data with different roles and objectives. The first observer focused on recording in detail the actions related to the handling, cooking, and storage of food, highlighting habits or behaviours related to exposure to microbiological and chemical risks in the drafting of the ethnographic notes. The second observer had a more dynamic role of more active participation, overseeing the collection of ethnographic notes related to the organisation and management of domestic spaces, using work tools, and observing family dynamics. In addition, photographic material was collected for each household regarding specific situations related to possible sources of risk in the kitchen, such as the arrangement of work surfaces, products, and resources both outside and inside refrigerators, freezers, cabinets, and drawers.

A total of six observers—social science researchers—took turns visiting the selected families. Before beginning observations, all researchers participated in training sessions on household food hazards held by a food safety expert. Protocols on how to collect data (diaries and photo) and how to interact with the subjects to make the observation system consistent were drawn up and shared.

A criterion used for the drafting of the diaries was that of separating the ethnographic notes into different levels: in the first level of description, an attempt was made to exercise the ‘thick descriptions’ [53], trying to use clean words and concepts tending towards minimal interpretation to capture the processuality of the phenomena; instead, the second level of description was modulated on the first form of understanding of the observational data, summarising sequences of actions through some concepts; finally, the last level of the diary reported the feelings and judgements of the observers themselves [54].

At the end of each observation, all the researchers involved in the activity took part in meetings to share their experience, discuss critical issues encountered, and develop common reflections on the approach to data collection and how to relate with the participants.

The ethnographic diaries were transcribed verbatim in digital format. A total of 28 diaries (2 diaries for each observation), 14 kitchen maps, 14 photographic sets of domestic refrigerators, and many photographs related to potential risk situations were collected.

Participants gave informed consent before taking part in the study. The study design meets the requirements established by the Ethics Committee of the Istituto Zooprofilattico Sperimentale delle Venezie.

### 2.3. Data Analysis

Ethnographic diaries were analysed by two social science researchers and a food safety researcher (food hygiene expert) through an interdisciplinary-oriented approach [25].

The analysis moved from an initial descriptive level to a conceptual level [21]. Categories were identified from the diaries produced by the observers, the analysis of which was conducted on two levels: in the first level, the practices related to microbiological and chemical risks were identified from the textual and visual data. Good strategies adopted by consumers and defensive behaviours were also highlighted. The second level concerned the analysis of risk perception, investigated through informal interviews during the observation sessions, which orientates the practices of the actors according to different logics and is not always based on avoiding microbiological and chemical hazards. The results presented here have focused primarily on the first level of analysis but still considering, in a cross-sectional manner, the second as well.

Although the different risk factors have been analysed separately, they are intertwined, generating a complex ecological system made of relationships that may or may not favour the incidence of food risks.

## 3. Results and Discussion

### 3.1. Overview of Practices and Factors That Facilitate Food-Related Risks

Practices and factors that facilitate food-related risks in the kitchen were identified through the data collected. The overview of the factors detected is presented in Table 2.

The detailed analysis of each identified risk factor is presented and discussed in the following paragraphs.

### 3.2. Factors That Facilitate Microbiological Risks

The data collected here highlight some practices and factors that facilitate food-related microbiological risk in the kitchen: inappropriate hand washing, presence of objects unrelated to food preparation in the kitchen spaces, improper use of dish towels and sponges, incorrect washing of utensils and food (such as eggs and vegetables), incorrect food storage, and presence of children and pets (Table 3).

#### 3.2.1. Inappropriate Hand Washing and Improper Handling of Food

The data collected show that the participants underestimated the fact that hands can be a vehicle for contamination and cross-contamination as they constitute the primary interface with the world. In this sense, hands are the most effective vehicle for transferring pathogenic microorganisms [1,27,55,56]. In many studies, respondents tended to declare that they adopted handwashing practices, although these were not implemented in the most appropriate ways [1,35,57].

In most families involved, meal preparers did not wash their hands before handling the food or before eating it, and they washed their hands after handling raw animal products such as meat (e.g., hamburgers), chicken breasts, and eggs. Hands were not washed after using the bathroom, sneezing, or blowing the nose and touching/stroking pets, dirty diapers, garbage, or objects unrelated to food preparation (e.g., mobile phones, remote controls, or hair clips dog bowls)—a factor observed in 13 out of 14 families. In several families, during meal preparation, those who prepared it had the habit of rinsing their hands frequently without using detergents. This practice was configured as a routine action and it was not clear when it was essential to do it and, above all, how to do it effectively to stop the possible transmission of pathogens and the consequent contamination of objects (e.g., dishcloths, utensils, or dishes) and foods. Family 1 showed problematic handling practices during the preparation of chicken, which was then fried—a practice that appears simple but requires particular attention to handling:

*After taking the chicken slices with her hands and putting them in a dish with breadcrumbs, the mother throws away the polystyrene package and rinses her hands. After that, she uses a tea towel to dry her hands, and the same tea towel is used several times to clean surfaces. […] During the preparation / breading of the chicken, the children help their mother by touching the ingredients with their hands without washing them. […] The child licks his hands after handling the raw chicken and offers his dad to lick him, too. The dad says, ‘It is not okay!’ The child stands up on the kitchen counter. The father then cleans, with his own hands, the breadcrumbs that came from breading the chicken.*

(Family 1)

In Family 2, a problematic handling of raw fish—followed by a lack of handwashing—was observed; raw fish is a source of pathogenic bacteria and washing fish in the sink without appropriate attention can favour their spread.

*The fish is taken from the fish market packaging by hand. Before starting this operation, I [the observer] did not notice if the mother washed her hands. After adding the breadcrumbs, she quickly rinses her hands without detergent. The fish was rinsed underwater in the sink before being placed in the pan. After this operation, the sink was not disinfected.*

(Family 2)

In Family 11, the handling of fish with latex gloves was observed, which allowed the hands to be isolated but did not replace handwashing. In this case, the mother declared in the interview that she did not like handling fish; therefore, the use of gloves may not have been dictated so much by microbiological concern but rather to mitigate disgust.

Not washing hands thoroughly after handling raw animal foods can expose consumers to the risk of contracting *Campylobacter*, *Salmonella*, and *Vibrio* infections [18,35,58]. It emerges from observational practices that participants significantly underestimate the presence of these two pathogens; thus, this underestimation does not facilitate the adoption of preventive behaviours that avoid their migration from raw meat, poultry, and fish to hands, cutting boards, salads, and taps [1].

Family 12, which included small children, showed several inattentions to hygiene practices that should be shared more within the family:

*‘It’s almost ready. Have you washed your hands?’ ‘Yes, yes’ replies the child. ‘No, you didn’t wash them’ says the dad. ‘But I washed them first!’ says the little girl. ‘Yes, but now you’ve touched things.’ She replies, ‘No, no, [points to the games] I didn’t touch!’ Dad argues with the daughter, tells her that she has to wash her hands. The little girl sitting at the table does not want to wash her hands. ‘Come on, then help me clear the table’, says the mother. […] ‘Did you wash your hands in the end?’ asks mom. But the little girl sits there, and in the end, she doesn’t wash her hands.*

(Family 12)

Observers have reported that hands are washed only after meals and not before, which indicates that the feeling of dirt is perceived as a result of the meal and the foul smell rather than due to the awareness of possible pathogens before consumption. This is in line with Wills’s work [21] in which cleaning—of hands or things—is not something that generally takes place as a discreet practice; the observed families often cleaned to tidy up or create a nice environment. The participants tended to focus on the superficial and ‘visual’ aspects of the domestic ‘dirt’ rather than on the aspects related to hygiene [18].

The use of detergents indeed reduces the microbial load present on the surfaces, resulting in ‘clean’ hands; however, these may not be microbiologically clean [59]. A suitable procedure would be to rub hands with water and soap for about 40–60 s to eliminate all microorganisms [60].

#### 3.2.2. Inappropriate Washing of Utensils, Work Surfaces and Food

Kitchen utensils (e.g., cutting boards and knives) and surfaces (e.g., worktops, table, and sink) can be vehicles for cross-contamination if not properly washed and sanitised after use [55], especially if they have come into contact with raw animal products or unwashed fruit and vegetables (some vegetables, such as onions, garlic, potatoes, are in close contact with the ground and therefore may have a higher bacterial load on the skin) [1,35]. Inappropriate washing of utensils, work surfaces, and food was observed in 11 out of 14 households.

In some families, the cutting board and other utensils were rinsed without special detergents and hot water, and conventional washing with cold water is ineffective [56]. In one family, the salad basket was placed on the sink compartment, where raw fish had been washed. The sink was then only rinsed and used to clean the salad (Family 2). The microbiological risks associated with the contamination of food prepared with dirty utensils previously used to prepare raw meat, poultry, and fish are particularly high [1,35].

**Figure 1:**
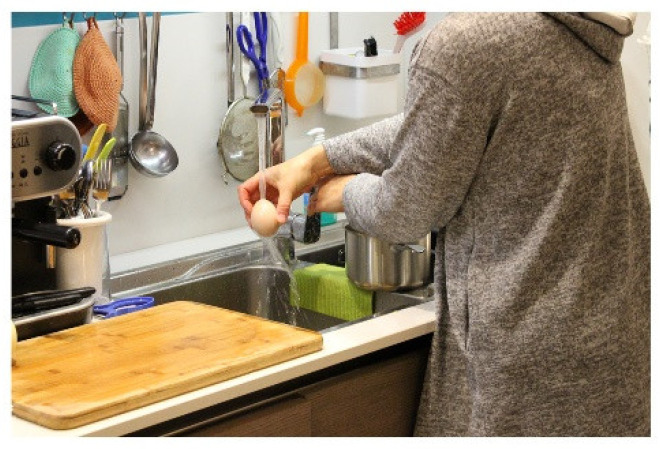
Egg washing.

The incorrect washing of the food itself can also promote cross-contamination. For example, various pathogenic microorganisms may be present on the eggshell, including *Salmonella* and *Escherichia coli* [61]. Washing eggs is not a correct operation because splashes of contaminated water could contaminate the sink and other kitchen surfaces as well as favour the penetration of microorganisms inside the egg, whose shell is porous (Figure 1).

*She takes the eggs, puts them near the sink, rinses them, and puts them in a pot. She touches the faucet and then two drawers to take the saucepan’s lid with her hands. She opens the garbage and throws away the potato skins. She rinses her hands only with water and dries herself.*

(Family 12)

Cooking is an excellent way to eliminate pathogenic microorganisms; the problem remains in the handling and non-washing of hands, which can recontaminate cooked food and other utensils. Fruits and vegetables may have traces of phytosanitary treatments on the peel or the external surface, or if they grow in close contact with the ground, residues of soil could potentially remain on the peel after harvesting (e.g., onions, garlic, potatoes, courgettes, pumpkins, melons, and watermelons) [21]. Family 8 failed to wash fruit and vegetable products:

*He takes three courgettes from the fridge (he does not wash them) and starts cutting them with the same knife as the onion and on the same cutting board; both tools have not been rinsed […] Then, he cleans the knife used for cutting the vegetables using the cloth hanging above the sink. Then, he takes a large piece of Parmesan cheese and cuts it into cubes with the same knife.*

(Family 8)

In Wills’s study [21], individuals who grew their fruit or vegetables were more likely to wash these products to remove perceived sand and dirt than those who bought them (apparently already clean). Since bacteria are invisible to the human eye, people cannot assess how clean the utensils and food are. Fruit and vegetables must be passed with running water to remove soil residues and pathogens, even if they are not perceived as dirty [21].

#### 3.2.3. Improper Use of Tea Towels, Dishcloths, and Sponges

Tea towels, dishcloths, and sponges are valuable devices for managing hygiene in the kitchen, but they can also become vehicles for microorganisms, favouring cross-contamination [1,55,62]. For example, after touching raw animal food, participants do not wash their hands with detergent and then dry the towels; afterward, the fabric is contaminated, similar to everything with which it enters into contact (e.g., surfaces, tools, hands, and food). Dishwashing sponges, counter towels, sinks, dishwashing trays, and bath sponges may contain high bacterial loads [34]. Improper use of towels and sponges was observed in 11 out of 14 families. In Family 8, which consisted of a pregnant wife and husband, observers noted that:

*There were three sponges: a steel scouring pad; a thin, soft pink sponge; and a two-layer, yellow-green, abrasive sponge. They say that ‘there is no real reason’ why they have two sponges and that apart from the straw, others are used in an undifferentiated way; ‘it is more because they accumulate there than for a real reason’.*

(Family 8)

Even sponges and dishcloths can collect and favour the proliferation of a microbial flora as varied as the variety of objects/surfaces with which they come into contact, especially if left wet at the bottom of the sink. Observers recorded that these objects often had no specific destination, and even when there was more than one, it did not always mean that the user respected the particular purpose for which the object was present. For example, the wife was convinced that some dishcloths were used only for the hands; then, the husband also used them to dry the sink and parts of the kitchen where the dog’s bowl had previously been placed (Family 13).

During Family 2’s dinner, several actions revolved around the use of tea towels, the handling of food, and the use of objects unrelated to the preparation of meals:

*The mother gets up and rinses her fingertips, dries them on the cloth hung on the door under the sink that contains the garbage, looks at her cell phone then sits down […]. Opening the door, the tea towel falls to the ground; she picks it up and puts it back on the door. The child takes the pear; ‘at least I’ll wash it’ she says, then rinses it and dries it in the towel stuck on the door under the sink. She hands it over to the son, who throws it on the floor, making vocalisations. […] He [the father] takes the leftovers in the sink with his hands, puts them in the trash, rinses his fingers, and dries them with a towel.*

(Family 2)

This sequence of actions describes shows how complex it can be to keep track of all the relationships involving practices, materiality, and pathogens.

**Figure 2:**
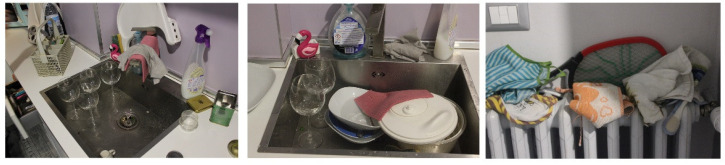
Management of sponges and tea towels.

The last photograph on the right (Figure 2) shows a radiator with several dishcloths, one of which was used to dry hands during the various stages of preparing dinner, tidying up the kitchen, and so on. After this sequence, the dishcloth was placed on top of the radiator alongside other things, including an electric scoop for mosquitoes and various bibs for babies (Family 2). The subject of the additional two photographs (Figure 2) is a pink sponge, which, after being collected from the sink containing dirty dishes, was used for different cleaning purposes without detergent.

Although handwashing is a good practice, its efficacy is inhibited by using inappropriate and previously contaminated tea towels and sponges. The proper management of these tools is essential for hygiene purposes—for example, not mixing tea towels for raw foods, hands, and surfaces and identifying suitable places to store these cleaning tools. Similarly, it is helpful to separate sponges for cleaning the house’s surfaces from those for cleaning dishes and pottery. The concept of separation, despite the initial efforts in terms of management, can become a procedure that hinders cross-contamination [21].

#### 3.2.4. Presence of Objects Unrelated to the Preparation of Food

**Figure 3:**
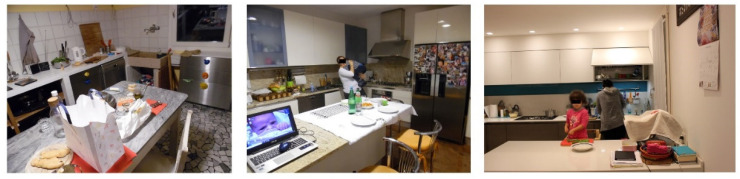
Objects unrelated to food handling.

In the kitchen, in addition to the tools useful for the preparation and processing of food, other objects that follow multiple paths of environmental exposure during the day make an appearance: for example, devices such as mobile phones, PCs, chargers, headphones, keys, wallets, and bags that accompany people everywhere in their routine, from public transport to work, the bar, and so on [1,63]. These objects unrelated to food preparation can be vehicles for pathogen microorganisms (Figure 3). In some families, the presence and use of cellular telephones during the preparation of meals, as well as remote controls, computers, tablets, books, games, and bags placed on the ground and then on the table or kitchen surfaces, were observed. Other items such as sprays, garbage buckets, hairpins, and clothespins for hair (*Staphylococcus aureus* can be present in people’s hair) were placed on the kitchen shelves. The presence of foreign objects unrelated to the handling of food in the kitchen was observed in 11 out of 14 families.

*During preparation, the kitchen shelves are crowded with objects (bottles, dishes used for cooking, utensils), and a plate containing parsley remains outside the fridge […] The father prepares the table, which appears clean with three placemats; on the table, there are a cellphone, a used glass, a battery charger hanging from the wall, and a basket with taralli.*

(Family 2)

The kitchen is not only the place where food is prepared but also a space in which multiple actors move and act in the area with different purposes (e.g., the mother cooks but at the same time also does homework with her daughter). The kitchen is a multifunctional open space where boundaries are blurred [21]; there is no clear separation between the space in which food is prepared and consumed and the one dedicated to other activities.

*At a certain point, the child arrives with papers and colours to finish her drawing. Mom tells her not to get on the table but to ‘get on the kitchen counter’, where, shortly before, there had been raw chicken. The little girl, standing, draws on the kitchen counter.*

(Family 1)

Separating places dedicated to preparation, consumption, and other activities would be a useful measure to avoid the relationships that facilitate cross-contamination [21]. There is a weak perception of the exposure paths of objects and that these connections can become the cause of cross-contamination.

#### 3.2.5. Problematic Conservation Practices

Preserving food in the best possible way (e.g., respecting the cold chain, placing food in the correct compartment of the refrigerator, avoiding leaving perishable food at room temperature for a long time, and not defrosting at room temperature) allows you to prevent the proliferation of pathogenic microorganisms [1,9].

The observers found different problems related to poor food storage and the improper use of storage devices in five families: food of animal origin was stored in the pantry in open containers, which were left at room temperature for a long time; fish products were left to thaw at room temperature for an unspecified time (Family 5); boiled, raw milk was left at room temperature (Family 9); finally, shopping bags—perhaps with perishable food inside—were left on the chair throughout the whole observation (Family 13).

**Figure 4:**
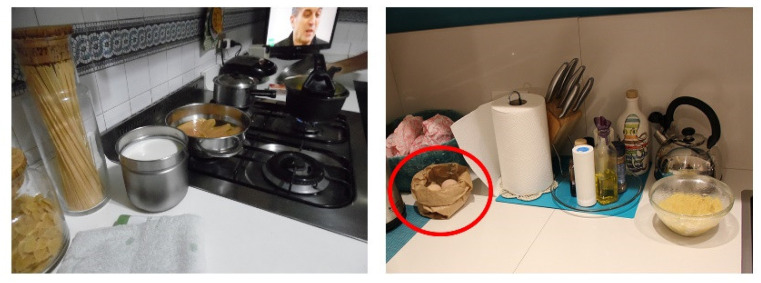
Raw milk and eggs left at room temperature.

In Figure 4, it is observed that the eggs are stored at room temperature in a basket on the kitchen surface—a practice confirmed through some questions asked by the observers to members of Family 12.

*She always keeps the eggs out. ‘I consume them within nine days; my mom goes to the chicken coop every day to get the eggs and writes the date on the shell. If I have to do raw stuff, I use them super fresh’.*

(Family 12)

Eggs are sold at room temperature to avoid the formation of condensation on the shell caused by the thermal shock that they would suffer if they were sold refrigerated [18,64]. Condensation facilitates the penetration of microorganisms inside the egg because the surface of the shell is porous. Once at home, however, the eggs must be kept in the refrigerator because low temperatures slow microbial growth. The eggs in Family 12 were rinsed underwater, which is also a risky practice as the egg is porous, and washing can favour the penetration of microbes from the shell.

**Figure 5:**
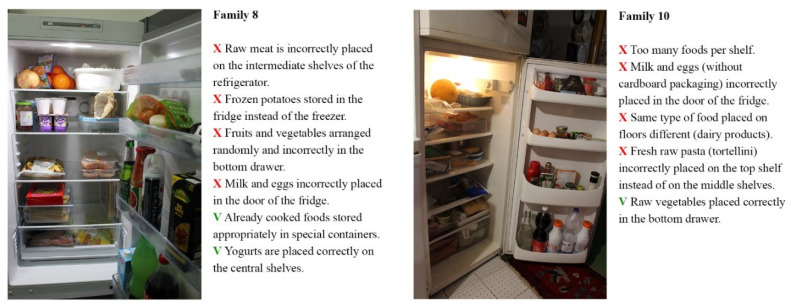
Evaluation of refrigerators.

For each family involved in the study, photographic material relating to the arrangement and storage of food in the refrigerator was collected. The analysis of visual materials was conducted by food safety experts who highlighted problematic aspects regarding the arrangement and methods of storing food inside them (Figure 5).

The refrigerator of Family 8 was well organised despite the following critical issues: raw meat incorrectly placed on the intermediate shelves, frozen potatoes stored in the fridge instead of in the freezer, fruit and vegetables arranged randomly and not correctly in the bottom drawer, and milk and egg products incorrectly placed in the door. In the refrigerator of Family 10, food arrangement also appeared to be quite random, and closed packages, open boxes, and plates with leftovers not covered by film were found.

Most of the photographs were judged negatively by the expert in food hygiene involved in the analysis, who highlighted that the arrangements and storage methods were not adequate for preventing the onset of food-related risks. From the study of the refrigerators, four problems emerged: the incorrect placement of food inside them, the arrangement of the same type of food on different floors, an excessive presence of food inside the various shelves, and drawers that could interfere with the automatic adjustment of the correct temperature (0–4°). Finally, an additional critical element is the non-use of specific sealed containers to preserve food already prepared and not wholly consumed.

Most of the families involved did not seem to pay particular attention to how food was arranged on the various shelves, and there was no rationality dictated by knowledge of the correct way of keeping food in the fridge. Instead, it followed the logic of random filling or the design of the refrigerator, whereby, for example, complementary foods were placed where there was a bottle holder or an egg holder. In Family 7, when asked to take photographs inside the fridge, a member warned the observers: ‘I have never observed a rule’, asking them for information on the correct positioning of the food (Family 7). Most of the subjects involved in the study did not seem to be aware that the refrigerator does not have a uniform temperature inside and that there are more or less cold areas in which it is advisable to store some foods rather than others depending on their specific characteristics and properties. This is in line with other research findings by which many consumers are unaware of proper refrigeration temperatures [1,65].

In addition to informing consumers about the correct use of their appliances, manufacturers should also be encouraged to design an internal refrigerator design that considers food safety rather than aesthetics; for example, using an appropriate symbol to help the consumer place the right food in the area with the ideal temperature for storage, inserting a small display for each frame showing the temperature, placing bottle holders for the milk in the refrigerator compartment and not only in the door (some fridges already have them), and removing egg holders from the door.

#### 3.2.6. Problematic Management of Pets and Children

**Figure 6:**
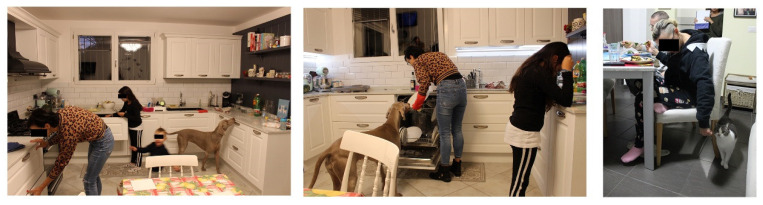
Management of pets in the kitchen.

The family ecology is conditioned by the presence of pets as they are considered part of the family, and particular hygiene attention is not paid when stroking them during lunch or allowing them to go wherever they want. In 3 out of 14 families, it was observed that the animals walked around the kitchen (Figure 6), coming into contact with the surfaces used to prepare food through their paws and with the dishes through the snout, by licking them.

*Before sitting down at the table, no one washed their hands. All the members are at the table; the baby is in his high-chair, where his mother helps him eat. The dog is free to move around them and is stroked/touched several times by the child and her father. The dog moves around the table, putting its muzzle on the table near the various diners, searching for food. […] At a certain point, the dog has its muzzle on the little girl’s plate. She asks if she can give chicken to the dog. The dog eats the chicken directly from the child’s plate, then she puts it back in the sink.*

(Family 1)

Physical contact between humans and pets can promote zoonotic diseases as animals carry pathogenic microorganisms that pose a risk to humans. In Family 8, a young pregnant woman explained her practices towards her cats:

*‘Cats are free to move anywhere but not on the table during meals.’ She, who is pregnant, strokes the cat as she passes by her chair during dinner.*

(Family 8)

Having animals can positively affect human health in psychological terms, but incorrect attention can have negative consequences on the family and the animal itself in terms of health [66,67].

In addition, the presence and behaviour of children can favour the possibility of contamination and cross-contamination of food. Observers mainly reported a case where one of the children stood up on the kitchen worktop and table, played with raw chicken breasts by putting his hands to his own mother and family members’ mouths, and tapped all over the place (Family 1). Children cross boundaries and experiment with the possibilities of a space, exploring and expanding it; even where there are rules, they show their agency and resist adults’ authority [21].

*I ask her if the children are always in the room or come to play in the living room. She says that ‘they tend to expand, but then they also stay in place’ […] actually, during the evening, the children seem very calm, polite, and disciplined, and even if they stay for periods of time in the kitchen, they do not create confusion but occupy a specific space at the back of the room on an armchair.*

(Family 7)

It is in families with young children that the circumstances in which cross-contamination or other food risks can occur increase as younger children require more attention, interactions, and contacts and transform the kitchen space into a multifunctional and multipurpose space [21,68]. For example, in Family 6, the observer noted that the presence of children interrupted the parents’ sequences of actions in the kitchen.

Parents are not always able to regulate access to the kitchen by gradually involving the youngest child; furthermore, the kitchen is generally perceived as a place of risk, not so much about food as to the physical dangers that can be incurred—primarily in the case of small children [21].

The mother is still on her knees; a few days ago, she broke a glass and tells us that she has gone into excessive paranoia. She has vacuumed a thousand times because the little girl crawls and puts everything in her mouth. She gets up, puts the broom and dustpan under the sink, and rinses her hands only with water. (Family 6)

*The mother turns to the little girl, who, in the meantime, has arrived in the kitchen. ‘Look at the little fish in the oven; this is a dangerous place’, she says. The grandmother comes to get the children and takes them back to the living room. […] The little girl arrives in the kitchen again and is sent away ‘You have to be with her grandmother; it’s dangerous here’.*

(Family 11)

Removing and separating the children from the kitchen is not the only suitable way to manage risks; for example, in Family 12, the mother was able to involve her daughter in preparing the meal:

*[The mother] offers the little girl to help her prepare dinner, asks her if she wants to wash the cabbage; ‘You have to involve them [children]’, she says. ‘Let us get down, so you help me. Are the hands clean?’ ‘Yes!’ replies the little girl. ‘Let me hear it […] No, they are not clean’ mom says, sniffing her little hands […]. They wash their hands with soap […]. The girl is cutting courgettes on a cutting board. The mother rinses the courgettes, cuts them lengthwise, and the little girl slices them.*

(Family 12)

In Family 7, there were ‘cooking rules’ posted above the stove in the kitchen. Undoubtedly, the children’s calmer and more polite behaviour can reduce the possibility of the kitchen becoming contaminated and chaotic. However, the rules of the game, the administration of spaces by adults, and the modulated forms of gradual involvement of children in the preparation of meals could prove fundamental means of avoiding microbiological risks as they promote forms of separation [1].

Even the distinction of ‘who does what’ roles can be functional to maintaining good hygiene practices. In families 2 and 14, a succession of actors underwent the same process: washing the salad, unloading the dishwasher, and not finding the fish lost in the kitchen. This uncoordinated but spontaneous and random overlap can introduce elements of risk as none of the actors is responsible for developing the process coherently and carefully in chaotic situations. Therefore, families where the adult agents divide up the tasks—where one of the two is dedicated to preparing the meal, while the other is in charge of looking after the children—are those in which the opportunities for risk decrease.

### 3.3. Factors That Facilitate Chemical Risks

The data collected show that some practices adopted in the kitchens can also increase food-related chemical risk; these practices include preserving food in unsuitable containers/materials, overcooking food, and being contaminated by detergents (Table 4).

#### 3.3.1. Food Storage in Unsuitable Containers/Materials

Storing food in containers unsuitable for contact with food or made with material unfit for that specific food may result in the migration of components of the material itself into the food and subsequently constitute a food risk of a chemical type [30]; an example is the use of some plastic tools at high temperatures and for purposes other than those recommended by the manufacturer [69]. The chemical risk varies depending on the substance involved and the amount ingested. These risk factors were found in 7 out of 14 families.

During the observations, the researchers highlighted that the containers or utensils used were not always adequate for contact with certain foods and high temperatures (e.g., hot chicken on plastic plates, or hot soup placed in a plastic container).

For example, it has been observed that some foods that are notoriously rich in salt (such as cheese and cured meats) or highly acidic have been preserved with aluminium foil despite this practice being discouraged by manufacturers. In other cases, such as in Family 9, some foods were stored directly in a pan placed in the refrigerator, but the material of pots is not always suitable for prolonged contact with food.

*She takes the cheese and three saucepans out of the fridge. We ask what is inside: one contains chicken slices, bought the day before, raw or only seared; in another one is pumpkin, pre-cooked; and the last contains courgettes, picked from a small back garden with her husband and pre-cooked.*

(Family 9)

The lady from Family 9 used cookware to store food in the fridge instead of containers and considered it something ‘that she has always done’. The tradition and ritual practices of yesteryear, which are handed down from generation to generation, may sometimes not align with current scientific knowledge.

#### 3.3.2. Overcooking of Food

**Figure 7:**
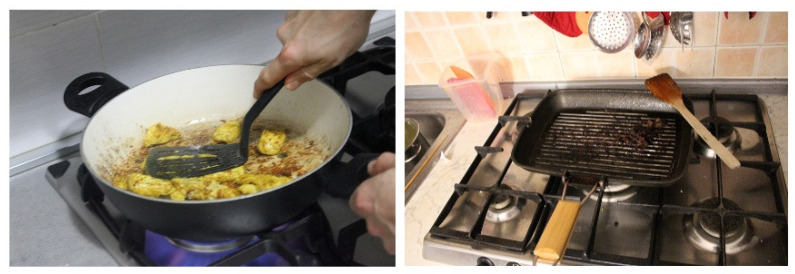
Burning meat.

Overcooking food can lead to chemical food hazards (Figure 7); in particular, meat and baked goods require specific care and attention during the cooking phase. Overcooking (up to burning) favours the development of carcinogenic substances such as polycyclic aromatic hydrocarbons (PAHs), heterocyclic amines, or acrylamide, which can pose a chemical risk [70]. This factor was found in 5 out of 14 families.

*I noticed that the cooking is speedy, over high heat, with consequent burning of the pans’ surfaces and the food, such as when sautéing, which I noticed from the fact that the onions used became black, and she put them aside as she ate. The pots seem heavily used, not well preserved, and usually washed in the dishwasher. Above the sink, there is an abrasive sponge used to scrape the bottom of the pots; they tell us that they use it precisely to clean food residues, which happens when food burns.*

(Family 8)

Even particular types of cooking—such as frying—require more attention than others.

*Then he turns on the pan for sautéing, adds some olive oil, takes some garlic, pops it out, and adds it to the oil. With the addition of garlic, the oil begins to gush and seems to be burning; he realises it and lowers the temperature.*

(Family 4)

The oil that burns and starts smoking represents a potential risk, as does its reuse for frying. A heated oil that visibly smokes means that it has reached its smoke point, which is the temperature at which the oil begins to burn and decompose, releasing visible volatile substances in the form of smoke and forming toxic or carcinogenic substances (for example, acrolein, PAHs [IPA], heterocyclic amines, acrylamides, nitrosamines, and so on) [70,71].

*They tell us about their reuse of the oil used to fry the chicken; they usually use it twice […] For frying, bottled (still sealed) olive oil is used; it is poured into a non-stick pan over high heat before dipping the meat … after dinner, the used oil is recovered and placed in a glass jar (without filtering it) to be reused once more.*

(Family 1)

Reusing oil is a habit acquired from the family of origin; oil had a cost, throwing it away was a waste, and new oil is a luxury that many families could not afford. This practice has remained—albeit with much information on the chemical risks associated with frying with used oil. Furthermore, there are frying devices available on the market that allow to reduce waste and control oil temperatures.

#### 3.3.3. Contamination by Detergents

Detergents are chemicals used to sanitise kitchen surfaces or utensils such as plates, glasses, or dishes. These are substances that should not be ingested because they can cause severe damage to the body. If the treated surfaces or utensils are not appropriately rinsed, or a residue of detergent remains on the bottom of the sink, the contact between residues of the detergent used and the food (e.g., the pasta drained in the sink whose drain is slow) can be a factor in food contamination [30,33]. This factor was found in only 1 out of 14 families. In one family, there was an episode in which the sink did not drain well, and the pasta came into contact with possible residues of detergents at the bottom of the sink.

*She goes back to the kitchen, takes a clean fork, tastes the pasta, says it lacks salt, and adds salt. […] She takes the colander, places it on the sink, takes the pasta pot, and drains the pasta. The sink fills with water. She says the sink gets clogged right away. She brings the drainer over the pot of turnip greens and tosses the pasta into the pan.*

(Family 4)

Implementing appropriate hygiene practices can virtually eliminate the risk of transmission of foodborne pathogens [56,62,72]. However, the excessive and incorrect use of some cleaning products can introduce another risk: the chemical contamination of food.

### 3.4. Practices Adopted by Families to Limit Food Risks

The data highlight some strategies adopted by the actors in the kitchen to protect themselves from foodborne diseases and particularly from microbiological risks. One of these is the use of senses such as sight and smell, through which we tend to evaluate the edibility of food in everyday life [21,38,73]. As shown by the observational data collected, some parents smell their children’s hands or visually and olfactively inspect food before cooking. This factor was observed in 4 out of 14 families (1, 2, 3, and 12).

*The mum goes back to the fish bag, opens it, lifts the trout fillets with her hands, looks at them, and puts them back down.*

(Family 2)

*She goes to open the chicken, which is wrapped in paper towels. She lifts the slices with her hands, looks at them, and then puts them back down.*

(Family 3)

Sight and smell are used to evaluate the edibility and quality of the food that is about to be consumed:

*The little girl sitting at the table cuts the chicken breast and says, ‘It is raw!’ ‘It cannot be’ her mother replies; ‘But inside, it is red’ the little girl says. ‘Because it is a tendon’ the mother answers. She eats the chicken.*

(Family 1)

In addition to the sense of smell, one relied on several other ways to assess the freshness of food, such as based on mould or whether the food ‘looked’ fresh in the refrigerator [38,73]. Moreover, in the study by Wills [21], attention was paid to sensory logic, which was used by the participants in the face of doubts and uncertainties related to the data placed on the label.

This type of action is related to the tendency to avoid food that caused illness; it is an emotion of disgust that constitutes a defence against microbial attacks and a protection from the risk of disease [18,74,75,76].

It is interesting to highlight that, although several elements contribute to facilitating microbiological risks, chemical risks emerge as a dominant concern in the interviews conducted during the observational sessions, which is in line with the findings of other studies [77,78,79]. Chemical dangers do not entirely depend on the individual behaviour of consumers but derive from pollution or companies that engage in fraudulent behaviour [29,80]. This type of risk falls within companies and control bodies; consumers, apart from buying through conventional sales channels and varying their diet, can do nothing when the contamination has already occurred before the purchase. Instead, while in the home, consumers can prevent chemical contamination caused by their actions—for example, by avoiding burning food. However, their fear of chemicals directs them to particular forms of supplying, preferring proximity and locality. What worries consumers most is the risks associated with dyes, preservatives, and pesticides [40,79,81]. In contrast, microbiological hazards seem to cause minor concern, although the practices connected to them are more frequent in the families observed.

For example, in Family 13, the mother addressed the provenance of the milk:

*But I prefer oat milk, rice milk, even almond milk, which is sweet; not soy milk because they say it is full of pesticides.*

(Family 13)

In the interviews, a dominant-negative evaluation of the products coming from industrial distribution which are considered less excellent and less healthy, emerges.

*He says that he does not like the vegetables of the supermarket because they are processed […] They tell us that the courgettes are from the garden that the husband takes care of, and the vegetables from the garden are eaten willingly. At the same time, they prefer not to buy vegetables from the supermarket because they are ‘processed’ but do not specify with what.*

(Family 9)

As shown by the dialogues, industrial production is often perceived as an obstacle to maintaining the quality of food, such as organic, locally produced, and self-produced food [82], as well as it is associated with chemical risk (preservatives, pesticides, and so on). However, in the absence of mediation and delegation to industry, some practices adopted at home can favour microbiological risks and decrease the nutritional properties of some foods—as in the case of Family 9, where the two older adults over 60 have the habit of buying raw milk from a farm; this product must undergo a boiling procedure to be consumed, as it is a possible source of pathogenic microorganisms. The participant’s ideas on how this boiling operation should be done are unclear; it is done ‘as it has always been done’. Still, higher temperatures (such as boiling temperatures) spoil the nutrients. Pasteurised (already processed) milk is more beneficial as it only reaches 72 °C for 15 s, eliminating only the pathogenic bacteria and preserving the nutritional principles as much as possible.

The participants mentioned preferring and adopting forms of self-production, buying supplies from neighbours, friends, and traditional shops. This factor was found in 6 out of 14 families (3, 7, 9, 10, 11, and 12).

*I get fruit and vegetables at the market near my house on Wednesdays and Saturdays. Virtually zero kilometres, and all grown in the countryside nearby! It is beautiful, also beautiful to look at! It is nice to go there early because they bring what they have, and the vegetables have a taste that you do not always find. *

(Family 3)

For example, Family 7 mentioned their preference for self-production of products such as tomato puree and jam:

*My mom always ate homemade tomato puree. Since I got married, it has been hard to get used to different tastes, and I did not know which packaged ones to buy … I tried to make tomato puree at home because it is tastier and healthier, and the tomatoes are from my mother’s garden, without preservatives or other substances … it is a matter of taste but also of the food’s healthy quality.*

(Family 7)

This devaluation of industrial production stimulates the tendency to orient to short and local food supply chains [32,83]; the participants believe that these foods are healthier and characterised by a more excellent nutritional value.

## 4. Conclusions

The methodological choice of ethnography made it possible to grasp the relationship between narratives, representations, and practices, highlighting their mutual implications. Furthermore, the collection of photographic material allowed understanding of not only what happens in the kitchen but also how the different rationalities that influence food practices translate into spaces’ organization and procedure of food management.

The results suggest that the adoption of appropriate food practices is not always related to risk knowledge. Even when there is awareness of the most appropriate behaviors to follow in the kitchen, the perception and management of risk are still shaped in relation to various forms of rationality and different scales of values, as highlighted by previous research [39].

In the present study, the most frequently found inadequacies are those related to microbiological risks. However, the interviews suggested that families’ main concerns are oriented towards the chemical dangers associated with industrial distribution, and this perception directs consumers towards local products and self-production [32].

The factors related to the microbiological risks found are incorrect handwashing, the presence in the kitchen spaces of objects unrelated to food preparation, the improper use of dishcloths and sponges, the inappropriate washing of utensils and food (such as eggs, fruit, and vegetables), the incorrect storage of food, and the presence of children and pets without regulating the use of spaces with material or symbolic separations.

The data show that in almost all the families observed, there was a lack of appropriate washing of hands with detergents, which is connected to the other factors as the hands, being the primary interface through which individuals come into contact with the environment, can act as a bridge for pathogenic organisms. This practice was often absent or replaced with frequent and quick rinsing and was conducted more for a clean and aesthetic feeling at the end of meals rather than to avoid microbiological risks, confirming results of international studies [18,21].

Moreover, little attention was given to the preservation of food and the maintenance of the cold chain; this could nullify the effect of the health surveillance actions performed in the food industry and the efforts implemented by sector operators to guarantee safe products through self-control and the Hazard Analysis and Critical Control Points (HACCP) system.

Practices that can expose consumers to chemical risk include food preservation through unsuitable containers/materials, overcooking food, and being contaminated by detergents. Risk factors related to the interaction dynamics between the different actors also emerged. More complex contexts and chaotic interactive dynamics can favour the emergence of risky situations—for example, the absence of separation of duties among actors preparing food; and the presence of animals and children, who require more attention and rules.

As observed in previous literature [6,21], the very structure of the physical space can orient the actions of individuals in such a way as to facilitate the adoption of certain practices, such as forms of material separation. For example, small and separate kitchens and spaces for children and pets enable a spontaneous division of roles. Even from a physical point of view, different, smaller spaces accompany the action and orient it without particular cognitive efforts; a dining table separated from the kitchen compartment, even through walls and doors, can make the dynamics more oriented towards separation and safety; however, good practices such as ‘cooking rules’ are necessary regardless of the design of the physical space available.

The methodological approach applied in this study allowed the highlighting of some critical issues that need further reflection both from a theoretical point of view and for the implementation of institutional communication strategies that should consider the situated, local, and contextual dimensions of food risks [24].

First, in many family contexts, it is recognizable the absence of a “culture of food safety” that can shape attitudes and practices aimed at food risk prevention and management at home. Often the ways in which actors behave in the kitchen reflect attitudes learned and passed down in the family context or practices based on “common sense”. Furthermore, as already noted in literature [73], the senses, in particular sight, touch, and smell, are among the main tools for identifying possible risk conditions. Scientific information, even when retrieved from scientific and institutional sources, struggles to be internalized and, therefore, to translate into correct habits.

Furthermore, the sensitivity toward food and nutrition claimed by some participants does not consistently translate into good practices. The relationship between eating habits and attention to health can provide a ground for the construction of risk communication strategies that consider the ambivalence between health concerns and new consumption styles [10].

In this regard, it is important to implement risk communication with information on food origin, traceability, and the functioning of the supply chain. This is even important from a risk prevention perspective since products perceived as similar may actually have different stories, methods of preparation, and conservation.

The results also suggest promoting the agency of the actors who move around the kitchen as a useful tool to prevent and manage risk situations. Encouraging the distribution of responsibilities among the actors, including children, can facilitate the learning of correct practices both for the handling of food as well as for the management of spaces and objects in the kitchen.

The data underline the need to implement communicative and training interventions that can provide precise and targeted indications and focus on hygiene rules (handwashing), the correct use of cook utensils (such as cutting boards, sponges, cutlery, and tea towels)—especially in their separation—and cooking practices. This is even more significant today with the COVID-19 pandemic because people have turned to preparing food at home more often.

However, it is clear that the dimension of risk is adapted and negotiated in relation to the social dynamics and relationships that occur in the kitchen. To be effective, risk communication strategies should consider the social and cultural aspects of food practices in the domestic environment [22,25].

## 5. Limitations

The ethnographic observation conducted in this research project was realized over days and hours to cope with certain limitations of time and resources available. However, this research design provides valuable information that would not be available without using these methods [84,85]. The data collected through this approach helped to understand contextual dimensions and explore the empirical results in greater depth. However, despite the advantages of observational methods, such as overcoming some biases linked to social desirability that sometimes invalidate the survey, the use of ethnography may entail some distortions such as the ‘saving face’ [86]: subjects who know that they are being observed tend to modify their actions based on perceived social desirability. In this research, the presence of observers may have influenced the families’ choice of menus (often quick and easy preparations such as chicken and salad) and therefore their performance. However, the distortions can be helpful because they can give those practices a heavier weight as things could be even more problematic in the absence of observers. The advantage is that some rituals and habits are deeply rooted in the family’s culture; patterns save cognitive energy and are therefore taken for granted. These habits are, by definition, difficult to modify and are not subject to question.

In addition, it would be interesting to repeat the study to verify whether the extensive information campaign implemented to stem the COVID-19 pandemic would have indirectly increased hygiene standards.

## Figures and Tables

**Table 1:** Characteristics of the observed sample.

Id	Family Components Present on Observation	Presence of Pets
Family unit: parents and children		
1	Mother (32 years), Father (35 years), 2 Children (9 e 2 years)	1 dog
2	Mother (44 years), Father (45 years), 2 Children (2 e 2 years)	
3	Mother (51 years), Father (52 years), 1 Child (14 years)	
4	Mother (50 years), Father (14 years), Grandpa (80 years)	1 cat
7	Mother (43 years), Father (40 anni), 2 Children (7 e 4 years)	
11	Mother (35 years), 2 Children (3 e 1 anno), Grandma	
12	Mother (31 years), Father (35 years), 1 Child (3 years)	
13	Mother (50 years), Father (54 years), 1 Child (13 years)	1 dog
14	Mother (40 years), Father (40 years), 2 Children (5 e 3 years)	
Family unit: presence of a pregnant woman		
6	Mother (41 years), Father (42 years), 1 Child (1 year)	
8	Mother (32 anni), Father (32 years)	2 cats, 1 snake
Family unit: couple over 60		
5	Wife (59 years), Husband (74 years)	
9	Wife (71 years), Husband (74 years)	
10	Wife (76 years), Husband (81 years)	1 cat

**Table 2:** Practices and factors that facilitate food-related risks and number of families in which they were observed.

Risks and Factors	Number of Families
*Microbiological risk*	
Inappropriate hand washing and improper handling of food	13
Inappropriate washing of utensils, work surfaces, and food	11
Improper use of tea towels and sponges	11
Presence of objects unrelated to the preparation of food	11
Problematic conservation practices	5
Problematic management of pets and children	3
*Chemical risk*	
Food storage in unsuitable containers/materials	7
Overcooking of food	5
Contamination by detergents	1

**Table 3:** Microbiological risk factors detected and families in which they were observed.

Factors Explored	Families ID
Inappropriate hand washing and improper handling of food	1, 2, 3, 4, 5, 6, 7, 8, 10, 11, 12, 13, 14
Inappropriate washing of utensils, work surfaces, and food	1, 2, 3, 5, 6, 8, 10, 11, 12, 13, 14
Improper use of tea towels and sponges	1, 2, 3, 6, 8, 9, 10, 11, 12, 13, 14
Presence of objects unrelated to the preparation of food	1, 2, 4, 5, 7, 8, 9, 11, 12, 13, 14
Problematic conservation practices	2, 4, 5, 9, 12
Problematic management of pets and children	1, 8, 13

**Table 4:** Factors that facilitate chemical risks in the families.

Factors Explored	Families ID
Food storage in unsuitable containers/materials	2, 3, 4, 8, 9, 12, 13
Overcooking of food	1, 4, 8, 10, 13
Contamination by detergents	4

## Data Availability

The data that support the findings of this study are available from the corresponding author upon reasonable request.

## References

[1] Byrd-Bredbenner C., Berning J., Martin-Biggers J., Quick V. (2013). Food safety in home kitchens: A synthesis of the literature. Int. J. Environ. Res. Public Health.

[2] European Food Safety Authority, European Centre for Disease Prevention and Control (2021). The European Union One Health 2019 Zoonoses Report. EFSA J..

[3] McLinden T., Sargeant J.M., Thomas M.K., Papadopoulos A., Fazil A. (2014). Component costs of foodborne illness: A scoping review. BMC Public Health.

[4] WHO Regional Office for Europe (2017). The Burden of Foodborne Diseases in the WHO European Region.

[5] World Health Organization (2015). WHO Estimates of the Global Burden of Foodborne Diseases: Foodborne Disease Burden Epidemiology Reference GROUP 2007–2015.

[6] Mihalache A.O., Møretrø T., Borda D., Dumitraşcu L., Neagu C., Nguyen-The C., Maître I., Didier P., Teixeira P., Junqueira L.O.L. (2022). Kitchen layouts and consumers’ food hygiene practices: Ergonomics versus safety. Food Control.

[7] Borrusso P.A., Quinlan J.J. (2017). Prevalence of pathogens and indicator organisms in home kitchens and correlation with unsafe food handling practices and conditions. J. Food Prot..

[8] Langiano E., Ferrara M., Lanni L., Viscardi V., Abbatecola A.M., De Vito E. (2012). Food safety at home: Knowledge and practices of consumers. Z. Gesundh. Wiss..

[9] Roccato A., Uyttendaele M., Membré J.M. (2017). Analysis of domestic refrigerator temperatures and home storage time distributions for shelf-life studies and food safety risk assessment. Food Res. Int..

[10] Cavazza N., Guidetti M. (2020). Scelte Alimentari: Foodies, Vegani, Neofobici e Altre Storie.

[11] Pellegrini G., Farinello F. (2009). Organic consumers and new lifestyles: An Italian country survey on consumption patterns. Br. Food J..

[12] Taché J., Carpentier B. (2014). Hygiene in the home kitchen: Changes in behaviour and impact of key microbiological hazard control measures. Food Control.

[13] Mascarello G., Pinto A., Marcolin S., Crovato S., Ravarotto L. (2017). Ethnic food consumption: Habits and risk perception in Italy. J. Food Saf..

[14] Cenci-Goga B., Amicabile A., Karama M., El-Ashram S., Saraiva C., García-Díez J., Finotti S., Genna V., Moretti G., Murari R. (2021). Effect of delayed refrigeration on the microbial carcass contamination of wild boars (Sus scrofa). Animals.

[15] Bloomfield S.F., Exner M., Fara M.G., Nath K.J., Scott E.A., Van der Voorden C. (2009). The Global Burden of Hygiene-Related Diseases in Relation to the Home and Community.

[16] Simmons D., Chapman G.E. (2012). The significance of home cooking within families. Br. Food J..

[17] Fonte M. (2002). Food systems, consumption models and risk perception in late modernity. Int. J. Sociol. Agric. Food.

[18] Møretrø T., Martens L., Teixeira P., Ferreira V.B., Maia R., Maugesten T., Langsrud S. (2020). Is visual motivation for cleaning surfaces in the kitchen consistent with a hygienically clean environment?. Food Control.

[19] Tirado C., Schmidt K. (2001). WHO surveillance programme for control of foodborne infections and intoxications: Preliminary results and trends across greater Europe. J. Infect..

[20] Griffith C., Worsfold D., Mitchell R. (1998). Food preparation, risk communication and the consumer. Food Control.

[21] Wills W.J., Meah A., Dickinson A.M., Short F. (2015). “I don’t think I ever had food poisoning”. A practice-based approach to understanding foodborne disease that originates in the home. Appetite.

[22] Fischer A.R.H., Frewer L.J. (2008). Food-safety practices in the domestic kitchen: Demographic, personality, and experiential determinants. J. Appl. Soc. Psychol..

[23] Røssvoll E.H., Ueland Ø., Hagtvedt T., Jacobsen E., Lavik R., Langsrud S. (2012). Application of hazard analysis and critical control point methodology and risk-based grading to consumer food safety surveys. J. Food Prot..

[24] Veflen N., Røssvoll E., Langsrud S., Scholderer J. (2020). Situated food safety behavior. Appetite.

[25] Fischer A.R.H., De Jong A.E.I., De Jonge R., Frewer L.J., Nauta M.J. (2005). Perspective: Improving food safety in the domestic environment: The need for a transdisciplinary approach. Risk Anal..

[26] Humphrey T.J., Martin K.W., Slader J., Durham K. (2001). *Campylobacter* spp. in the kitchen: Spread and persistence. J. Appl. Microbiol..

[27] Mattick K.L., Bailey R.A., Jørgensen F., Humphrey T.J. (2002). The prevalence and number of *Salmonella* in sausages and their destruction by frying, grilling or barbecuing. J. Appl. Microbiol..

[28] Beck U. (1992). Risk Society: Towards a New Modernity.

[29] Tonkin E., Coveney J., Meyer S.B., Wilson A.M., Webb T. (2016). Managing uncertainty about food risks—Consumer use of food labelling. Appetite.

[30] Rather I.A., Koh W.Y., Paek W.K., Lim J. (2017). The sources of chemical contaminants in food and their health implications. Front. Pharmacol..

[31] Prati G., Cicognani E., Cicognani E., Prati G., Zani B. (2011). Percezione e comunicazione del rischio: Uno sguardo alla letteratura. Uranio Impoverito. Percezione e Comunicazione Del Rischio.

[32] Tonkin E., Webb T., Henderson J., Ward P.R., Coveney J., Meyer S.B., McCullum D., Wilson A.M. (2021). The health implications of distrust in the food system: Findings from the dimensions of trust in food systems scale (DOTIFS scale). BMC Public Health.

[33] Hawkes G., Rowe G. (2008). A characterisation of the methodology of qualitative research on the nature of perceived risk: Trends and omissions. J. Risk Res..

[34] Ojima M., Toshima Y., Koya E., Ara K., Tokuda H., Kawai S., Kasuga F., Ueda N. (2002). Hygiene measures considering actual distributions of microorganisms in Japanese households. J. Appl. Microbiol..

[35] Redmond E.C., Griffith C.J. (2003). Consumer food handling in the home: A review of food safety studies. J. Food Prot..

[36] Rusin P., Orosz-Coughlin P., Gerba C. (1998). Reduction of faecal coliform, coliform and heterotrophic plate count bacteria in the household kitchen and bathroom by disinfection with hypochlorite cleaners. J. Appl. Microbiol..

[37] Kendall H., Brennan M., Seal C., Ladha C., Kuznesof S. (2016). Behind the kitchen door: A novel mixed method approach for exploring the food provisioning practices of the older consumer. Food Qual. Prefer..

[38] Martens L., Pink S. (2012). The Politics and Practices of Looking: CCTV Video and Domestic Kitchen Practices. Advances in Visual Methodology.

[39] Meah A. (2014). Still blaming the consumer? Geographies of responsibility in domestic food safety practices. Crit. Public Health.

[40] Meah A., Watson M. (2011). Saints and slackers: Challenging discourses about the decline of domestic cooking. Sociol. Res. Online.

[41] Mihalache O.A., Dumitraşcu L., Nicolau A.I., Borda D. (2021). Food safety knowledge, food shopping attitude and safety kitchen practices among Romanian consumers: A structural modelling approach. Food Control.

[42] Møretrø T., Nguyen-The C., Didier P., Maître I., Izsó T., Kasza G., Skuland S.E., Cardoso M.J., Ferreira V.B., Teixeira P. (2021). Consumer practices and prevalence of *Campylobacter*, *Salmonella* and norovirus in kitchens from six European countries. Int. J. Food Microbiol..

[43] Rotheram S., Cooper J., Barr B., Whitehead M. (2021). How are inequalities generated in the management and consequences of gastrointestinal infections in the UK? An ethnographic study. Soc. Sci. Med..

[44] Sutton D. (2009). The Mindful Kitchen, The Embodied Cook: Tools, Technology and Knowledge Transmission on a Greek Island.

[45] Brennan M., McCarthy M. (2016). Food Handling in the Home.

[46] Halkier B., Jensen I. (2011). Methodological challenges in using practice theory in consumption research. Examples from a study on handling nutritional contestations of food consumption. J. Consum. Cult..

[47] Trappmann M., Krumpal I., Kirchner A., Jann B. (2014). Item sum: A new technique for asking quantitative sensitive questions. J. Surv. Stat. Methodol..

[48] Simon H.A. (1955). A Behavioral Model of Rational Choice. Q. J. Econ..

[49] Corbetta P. (2003). Social Research: Theory, Methods and Techniques.

[50] Glaser B.G., Strauss A.L. (1967). The Discovery of Grounded Theory: Strategies for Qualitative Research.

[51] Hammersley M., Atkinson P. (1995). Ethnography: Principles in Practice.

[52] Bateson G. (1993). Verso Un’ecologia Della Mente.

[53] Geertz C. (1973). The Interpretation of Cultures: Selected Essays.

[54] Sclavi M. (2003). Arte Di Ascoltare e Mondi Possibili: Come Si Esce Dalle Cornici Di Cui Siamo Parte.

[55] Evans E.W., Redmond E.C. (2019). Domestic kitchen microbiological contamination and self-reported food hygiene practices of older adult consumers. J. Food Prot..

[56] Cogan T.A., Bloomfield S.F., Humphrey T.J. (1999). The effectiveness of hygiene procedures for prevention of cross-contamination from chicken carcases in the domestic kitchen. Lett. Appl. Microbiol..

[57] Clayton D.A., Griffith C.J., Price P., Peters A.C. (2002). Food handlers’ beliefs and self-reported practices. Int. J. Environ. Health Res..

[58] Mandal R., Shi Y., Singh A., Yada R.Y., Pratap Singh A. (2020). Food Safety and Preservation. Biomed Sci..

[59] Gibson L.L., Rose J.B., Haas C.N., Gerba C.P., Rusin P.A. (2002). Quantitative assessment of risk reduction from hand washing with antibacterial soaps. J. Appl. Microbiol. Symp. Suppl..

[60] World Health Organization, WHO Patient Safety (2009). WHO Guidelines on Hand Hygiene in Health Care.

[61] Luber P. (2009). Cross-contamination versus undercooking of poultry meat or eggs—Which risks need to be managed first?. Int. J. Food Microbiol..

[62] Kusumaningrum H.D., Riboldi G., Hazeleger W.C., Beumer R.R. (2003). Survival of foodborne pathogens on stainless steel surfaces and cross-contamination to foods. Int. J. Food Microbiol..

[63] Redmond E.C., Griffith C.J. (2009). The importance of hygiene in the domestic kitchen: Implications for preparation and storage of food and infant formula. Perspect. Public Health.

[64] Whiley H., Clarke B., Ross K. (2017). Knowledge and attitudes towards handling eggs in the home: An unexplored food safety issue. Int. J. Environ. Res. Public Health.

[65] Lange M., Göranzon H., Marklinder I. (2016). Self-reported food safety knowledge and behaviour among Home and Consumer Studies students. Food Control.

[66] Cloutier A., Peetz J. (2016). Relationships’ best friend links between pet ownership, empathy, and romantic relationship outcomes. Anthrozoos.

[67] Krueger W.S., Hilborn E.D., Dufour A.P., Sams E.A., Wade T.J. (2016). Self-Reported Acute Health Effects and Exposure to Companion Animals. Zoonoses Public Health.

[68] Byrd-Bredbenner C., Maurer J., Wheatley V., Cottone E., Clancy M. (2007). Food safety hazards lurk in the kitchens of young adults. J. Food Prot..

[69] Ehlert K., Beumer C. (2008). Migration study of bisphenol A into water from polycarbonate baby bottles during microwave heating. Food Addit. Contam..

[70] Nerín C., Aznar M., Carrizo D. (2016). Food contamination during food process. Trends Food Sci. Technol..

[71] Roccato A., Uyttendaele M., Cibin V., Barrucci F., Cappa V., Zavagnin P., Longo A., Ricci A. (2015). Survival of *Salmonella Typhimurium* in poultry-based meat preparations during grilling, frying and baking. Int. J. Food Microbiol..

[72] Kusumaningrum H.D., Paltinaite R., Koomen A.J., Hazeleger W.C., Rombouts F.M., Beumer R.R. (2003). Tolerance of *Salmonella Enteritidis* and *Staphylococcus aureus* to Surface Cleaning and Household Bleach. J. Food Prot..

[73] Young I., Waddell L. (2016). Barriers and facilitators to safe food handling among consumers: A systematic review and thematic synthesis of qualitative research studies. PLoS ONE.

[74] Curtis V., Aunger R., Rabie T. (2004). Evidence that disgust evolved to protect from risk of disease. Proc. R. Soc. B Biol. Sci..

[75] Fernandes N.L., Pandeirada J.N.S., Soares S.C., Nairne J.S. (2017). Adaptive memory: The mnemonic value of contamination. Evol. Hum. Behav..

[76] Nemeroff C., Rozin P. (1994). The Contagion Concept in Adult Thinking in the United States: Transmission of Germs and of Interpersonal Influence. Ethos.

[77] Buchler S., Smith K., Lawrence G. (2010). Food risks, old and new: Demographic characteristics and perceptions of food additives, regulation and contamination in Australia. J. Sociol..

[78] Saleh R., Bearth A., Siegrist M. (2019). “Chemophobia” Today: Consumers’ Knowledge and Perceptions of Chemicals. Risk Anal..

[79] Kher S.V., De Jonge J., Wentholt M.T.A., Deliza R., de Andrade J.C., Cnossen H.J., Luijckx N.B.L., Frewer L.J. (2013). Consumer perceptions of risks of chemical and microbiological contaminants associated with food chains: A cross-national study. Int. J. Consum. Stud..

[80] Beck U. (2009). World at Risk.

[81] Bruhn C.M., Schutz H.G. (1999). Consumer Food Safety Knowledge and Practices. J. Food Saf..

[82] Mascarello G., Pinto A., Parise N., Crovato S., Ravarotto L. (2015). The perception of food quality. Profiling Italian consumers. Appetite.

[83] Ekici A., Kahn B.E., Luce M.F. (2004). Consumer Trust and Distrust in the Food System: Some Implications for the Debates on Food Biotechnologies. Advances in Consumer Research.

[84] Isaacs E., Jordan B. (2013). The value of rapid ethnography. Advancing Ethnography in Corporate Environments: Challenges and Emerging Opportunities.

[85] Moses N.D., Pakravan M.H., MacCarty N.A. (2019). Development of a practical evaluation for cookstove usability. Energy Sustain. Dev..

[86] Goffman E., Best J. (1967). Interaction Ritual: Essays in Face-to-Face Behavior.

